# Cardiac resynchronization therapy guided by cardiovascular magnetic resonance

**DOI:** 10.1186/1532-429X-12-64

**Published:** 2010-11-09

**Authors:** Francisco Leyva

**Affiliations:** 1 Centre for Cardiovascular Sciences, Queen Elizabeth Hospital, University of Birmingham, UK

## Abstract

Cardiac resynchronization therapy (CRT) is an established treatment for patients with symptomatic heart failure, severely impaired left ventricular (LV) systolic dysfunction and a wide (> 120 ms) complex. As with any other treatment, the response to CRT is variable. The degree of pre-implant mechanical dyssynchrony, scar burden and scar localization to the vicinity of the LV pacing stimulus are known to influence response and outcome. In addition to its recognized role in the assessment of LV structure and function as well as myocardial scar, cardiovascular magnetic resonance (CMR) can be used to quantify global and regional LV dyssynchrony. This review focuses on the role of CMR in the assessment of patients undergoing CRT, with emphasis on risk stratification and LV lead deployment.

## Introduction

The first demonstration of a beneficial effect from cardiac resynchronization therapy (CRT) was provided by Cazeau et al who, in 1994, treated a 54 year old heart failure patient with four-chamber pacing [1]. Subsequently, acute hemodynamic studies showed that CRT improves cardiac output [2,3]. The Multisite Stimulation in Cardiomyopathies (MUSTIC) study demonstrated that CRT led to an improvement in NYHA class, quality of life, exercise capacity and peak oxygen uptake, as well as to a reduction in heart failure hospitalizations [4]. By 2005, the Cardiac Resynchronization in Heart Failure (CARE-HF) study of patients with moderate-to-severe heart failure showed that CRT-pacing (CRT-P) led to a 36% relative reduction in total mortality [5]. The Comparison of Medical Therapy, Pacing and Defibrillation in Heart Failure (COMPANION) study, which included a similar patient group, had previously shown that addition of a cardioverter defibrillator (CRT-D) led to an additional survival benefit [6]. More recently, the Multicenter Automatic Defibrillator Implantation Trial with Cardiac Resynchronization Therapy (MADIT-CRT) trial has shown that compared with implantable cardioverter defibrillators (ICD) therapy alone, CRT-D therapy is associated with a dramatic reduction in the risk of heart-failure events in relatively asymptomatic (NYHA class I and II) ICD recipients with a low LVEF and wide QRS complex [7]. The Resynchronization Reverse Remodelling in Systolic Left Ventricular Ventricular dysfunction (REVERSE) study has shown that CRT leads to LV reverse remodelling and an increase in LVEF in the context of milder symptoms (NYHA class I/II) [8]. Studies are already underway to explore the possible benefits of CRT in patients with conventional indications for pacing [9] and patients with a narrow QRS duration [10]. Even if only one of these new indications is added to current guidelines (Tables 1**and **2), the demand for CRT is likely to increase exponentially over the current decade.

**Table 1:** European Society of Cardiology guidelines for cardiac resynchronization therapy (2010 Update).*

Recommendation	Patient population	Class	Level
			

**Sinus rhythm**			

CRT preferentially by CRT-D is recommended to reduce morbidity or to prevent disease progression	NYHA class II	I	A
	LVEF ≤ 35%, QRS ≥ 150 ms		

CRT-P/CRT-D is recommended to reduce morbidity and mortality	NYHA class III/IV	I	A
	LVEF ≤ 35%, QRS ≥ 120 ms		
	Optimal medical therapy		

			

**Atrial fibrillation**			

CRT-P/CRT-D should be considered to reduce morbidity	NYHA class III/IV	IIa	B
	LVEF ≤ 35%, QRS ≥ 130 ms		
	Permanent dependency induced by AV nodal ablation		

CRT-P/CRT-D should be considered to reduce morbidity	NYHA class III/IV	IIa	C
	LVEF ≤ 35%, QRS ≥ 130 ms		
	Slow ventricular rate and frequent pacing		

			

**Concomitant Class I pacemaker indication**			

CRT-P/CRT-D is recommended to reduce morbidity	NYHA class III/IV	I	B
	LVEF ≤ 35%, QRS ≥ 120 ms		

CRT-P/CRT-D is recommended to reduce morbidity	NYHA class III/IV	IIa	C
	LVEF ≤ 35%, QRS < 120 ms		

CRT-P/CRT-D is recommended to reduce morbidity	NYHA class II	IIb	C
	LVEF ≤ 35%, QRS < 120 ms		

*Recommendations according to presence of sinus rhythm, atrial fibrillation or concomitant conventional pacemaker indications, taken from Dickstein K, et al. [126] Class and level of evidence is shown in the columns. CRT-P = cardiac resynchronization therapy pacing; CRT-D = cardiac resynchronization therapy - defibrillation.

**Table 2:** American College of Cardiology/American Heart Association/Heart Rhythm Society guidelines for cardiac resynchronization therapy (2008).*

Recommendation	Patient population	Class *
CRT-D/CRT-P	NYHA Class III/IV	I
	LVEF ≤ 35%, SR	
	QRS ≥ 120 ms	
	Optimal medical therapy	

CRT-D/CRT-P	NYHA Class III/IV	IIa
	LVEF ≤ 35%,	
	Optimal medical therapy	
	Frequent dependence on	
	ventricular pacing	

CRT-D/CRT-P	NYHA Class III/IV	IIa
	LVEF ≤ 35%	
	QRS ≥ 120 ms	
	Optimal medical therapy	
	Atrial fibrillation	

CRT-D/CRT-P	NYHA Class I or II	IIb
	LVEF ≤ 35%	
	Optimal medical therapy	
	Dvice implant with anticipated	
	frequent ventricular pacing	

*Recommendations taken from Epstein AE, et al. [127] Class and level of evidence, according to ACC/AHA/HRS classification, is shown in the column. CRT-P = cardiac resynchronization therapy pacing; CRT-D = cardiac resynchronization therapy - defibrillation.

Appropriate diagnosis and management of heart failure not only involves an accurate assessment of myocardial and valvular function, but also of heart failure etiology. Additional aspects that are relevant to CRT are mechanical dyssynchrony, scar burden and scar localization in the vicinity of the LV pacing stimulus. With its ability to provide a one-stop assessment of all these aspects of cardiac structure and function, cardiovascular magnetic resonance (CMR) is gaining credence as a routine imaging modality for patients undergoing CRT. This review focuses on the available evidence for using CMR in the diagnostic work-up and implantation in patients undergoing CRT. The potential for further development is also explored.

### Responders and non-responders to CRT

The terms 'responder' and 'non-responder' are frequently used in relation to CRT. Yet, there is no consensus on what should be considered a response [11]. Some authors use clinical variables, such as an improvement in NYHA class or walking distance, whereas others use composite measures, such as freedom from hospitalizations or left ventricular reverse remodelling. Whilst the notion of response is conceptually attractive, we should consider the response rate to pharmacological therapy for heart failure. For example, an improvement in ≥ 1 NYHA classes is only observed in 46.7% of patients treated with enalapril, [12] 21% of patients treated with bisoprolol[13] and 41% of patients treated with spirinolactone [14]. In relation to placebo, the responder rates with these drugs are 24.9% for enalapril, 6% for bisoprolol and 8% for spironolactone.

If we are to use 'response' in managing patients undergoing CRT, composite measures are probably the most useful. The combination of changes in NYHA class and LV reverse remodelling, for example, is easily quantifiable and clinically useful. We should consider, however, that the lack of a symptomatic benefit does not necessarily imply absence of a prognostic benefit.

### Dyssynchrony as the target in CRT

Conducting tissue disturbances give way to conduction through the slower-conducting myocardium, delays in ventricular activation, wasted work, [15] a reduction in cardiac output [16] and LV end-systolic dilatation [17]. According to the most popular paradigm of CRT, cardiac dyssynchrony arising from such disturbances contributes to the syndrome of heart failure and its correction leads to a clinical benefit. This paradigm, which dictates that pre-implant dyssynchrony is a *sine qua non *for a benefit from CRT, has driven the unrelenting search for a dyssynchrony measure as a predictor of response to and outcome of CRT.

#### Echocardiography

In the pursuit for a reliable CMR measure of dyssynchrony, we must consider the extensive body of evidence in relation to dyssynchrony and CRT provided by echocardiography. Amongst the earliest and the simplest echocardiographic measures of dyssynchrony to emerge was the septal-to-posterior wall motion delay, [18] which is the absolute time difference between peak septal and peak posterior wall motion towards the centre of the LV. Amongst the most complex is the absolute difference or the standard deviation of the time-to-peak systolic wall motion on tissue Doppler imaging in various (usually 12) myocardial segments [19]. These and multiple other measures raised great expectations as predictors of response to and outcome of CRT in early single-centre studies [18,20,21]. Their utility were further tested in the Predictors of Response to CRT (PROSPECT) trial, a multicentre study in which 12 echocardiographic parameters of dyssynchrony were assessed by a blinded core laboratory in 498 patients with standard CRT indications [22]. In this study, the ability of echocardiographic measures to predict clinical response varied widely, with sensitivities ranging from 6% to 74% and receiver-operator characteristics curves of ≤ 0.62. Importantly, the interobserver coefficient of variation for these measures were as high as 72.1% for the septal-to-posterior wall motion delay and 33.7% for the standard deviation of the time-to-peak systolic wall motion of 12 myocardial segments on tissue Doppler imaging [22]. Essentially, the PROSPECT study found that no single echocardiographic measure of dyssynchrony improved patient selection for CRT beyond current guidelines. In CARE-HF, [5] the first and only randomized controlled study of CRT to include measures of dyssynchrony in patients selection, no single echocardiographic measure of dyssychrony emerged as a clinically applicable predictor of outcome. After much expectation, echocardiographic measures of dyssynchrony have not gained credence in selecting patients for CRT [23-26].

#### Normal QRS duration

The purist view is that there is no treatable dyssynchrony at a QRS < 120 ms and an LVEF > 35%. Dyssynchrony, however, is a biological phenomenon and as such, is expected to behave as a continuous rather than as a dichotomous variable [27,28]. Not surprisingly, therefore, cardiac dyssynchrony is detectable in patients with a QRS ≤ 120 ms [29-35] and higher LVEFs [36,37]. Using CMR, we have shown that up to 91% of patients with a QRS < 120 have radial dyssychrony [38]. (Additional File 1; **Movie 1**) It seems reasonable to suppose, therefore, that at least some patients with a QRS < 120 ms might benefit from CRT. Although several observational studies have reported a benefit in patients with a QRS < 120 ms, [39-41] the recently reported Resynchronization Therapy in Narrow QRS (RethinQ) study, a randomized controlled trial, showed no benefit in terms of peak oxygen consumption [42]. This study, however did not use an implantation technique that avoids pacing LV scar. Whether or not CMR-guided CRT (avoiding scar) is effective in patients with a QRS < 120 ms remains to be explored.

#### Does the magnitude of pre-implant dyssynchrony affect the CRT response?

**Figure 1:**
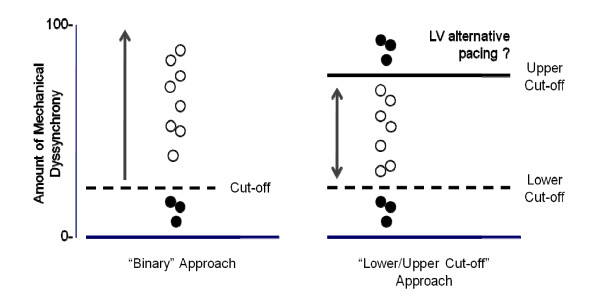
**Paradigms in cardiac resynchronization therapy**. Schematic representation of two paradigms of mechanical dyssynchrony in cardiac resynchronization therapy. The left panel shows a "binary approach", according to which mechanical dyssynchrony above a certain cut-off is associated with a benefit from CRT (open circles), whereas dyssynchrony below that cut-off is associated with no benefit (solid circle). The right panel depicts the "lower/upper cut-off approach", according to which patients within a finite range of mechanical dyssynchrony benefit from CRT (open circles), whereas patients with too much dyssynchrony (above the upper cut-off) or too little dyssynchrony (below the lower cut-off) do not benefit (closed circles). It is hypothesized that, outside the critical range, alternative pacing modes (endocardial pacing, LV pacing with multiple leads) may be more effective than conventional CRT. Reproduced with permission from Auricchio and Leyva [128].

According to the currently accepted paradigm of CRT, dyssynchrony contributes to the syndrome of heart failure and its correction by CRT leads to a clinical benefit. The question arises as to what degree of dyssynchrony is reversible by CRT. In this respect, we have found that and that the most severely scarred LVs are the most dyssynchronous [38] and that extremes of dyssynchrony relate to a high mortality and morbidity after CRT [34] These findings suggest that there are extremes of dyssynchrony that cannot be corrected by CRT (Figure 1)[43].

### Assessing dyssynchrony with CMR

Except for the UK NICE, [44] no guideline group has adopted a criterion of mechanical dyssynchrony in relation to CRT. Importantly, NICE has not specified which measure of mechanical dyssynchrony should be adopted, nor what cut-off to apply. In the absence of a consensus, therefore, measures of mechanical dyssynchrony cannot be used to decide on eligibility for CRT, even in the UK. The question remains, however, as to whether measures of mechanical dyssynchrony can be used for risk stratification and for guiding LV lead deployment.

#### Steady-state free precession (SSFP)

**Figure 2:**
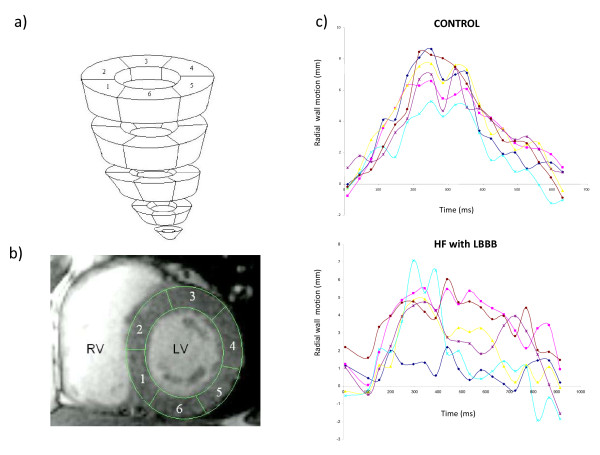
**Wall motion analysis using SSFP sequences**. Wall motion analysis using SSFP sequences involves division of the left ventricular myocardium into slices and segments (a). The latter extend clockwise from the junction between the interventricular septum and the right ventricular free wall (beginning of segment 1 and end of segment 6) (b). As shown in (c), the concordance of radial wall motion observed in healthy control subjects is highly disrupted in patients with heart failure and a left bundle branch block (LBBB).

**Figure 3:**
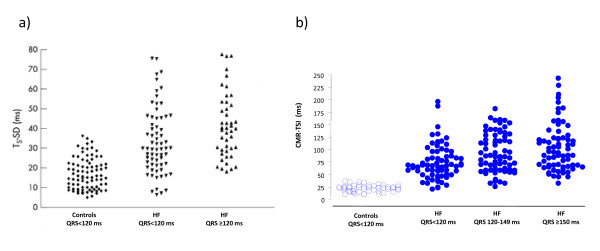
**Echocardiographic and CMR measures of dyssynchrony in heart failure**. Distribution of dyssynchrony measures obtained from echocardiography and CMR. The standard deviation of the time to peak myocardial sustained systolic velocity (Ts-SD) of 12 left ventricular segments (a) and the CMR-tissue synchronization index CMR-TSI) (b) in controls and in patients with heart failure (HF), stratified according to QRS duration. Note the considerable overlap between healthy controls and patients with heart failure. Adapted with permission from a) Yu C-M et al [30] and (b) Foley et al. [38]

**Figure 4:**
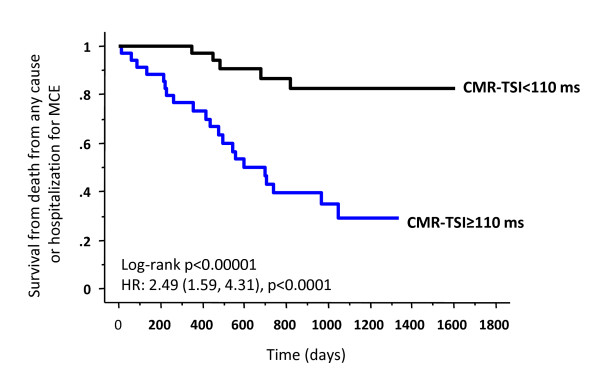
**CMR-TSI and outcome of CRT**. Kaplan-Meier estimates of the time to the composite endpoint of death from any cause or hospitalization for a major cardiovascular event (MCE) after CRT.. Patients were stratified according to a pre-implant CMR-TSI < 110 ms or CMR-TSI ≥ 110 ms. Results of univariate Cox proportional hazards analyses are expressed in terms of the hazard ratio (HR) and 95% confidence limits. Reproduced with permission from Chalil S, et al. [34]

This 'work-horse' CMR sequence provides a wealth of anatomical and functional information that can be used to derive measures dyssynchrony [34,45]. We have used conventional SSFP short-axis stacks and semi-automatic manual planimetry to quantify radial wall motion from atrioventricular ring to apex. (Figure 2) [34]. Using commercial software (MASS analysis software, Medis, The Netherlands) each short-axis slice is divided into cords running circumferentially around the LV. Radial wall motion is quantified semi-automatically for up to 20 phases in each R-R interval. The time-dependent segmental radial wall motion curve is then smoothed by fitting to an empirical sine wave. The CMR tissue synchronization index (CMR-TSI) is then calculated as the standard deviation of all segmental phase shifts of the radial wall motion extracted from the fit. As shown in Figure 3, this technique reveals that dyssynchrony is almost universal in patients with heart failure [38] and that the CMR-TSI is a better at discriminating between patients with heart failure and healthy control subjects than echocardiographic measures of dyssynchrony [38]. Moreover, the CMR-TSI provides prognostic information. In a long-term follow-up of 77 patients undergoing CRT, those with a CMR-TSI ≥ 110 ms were more likely to meet the endpoints of death from any cause or hospitalization for a major adverse cardiovascular event (Figure 4), death from any cause or hospitalization for heart failure, and death from cardiovascular causes from cardiovascular causes than those with a CMR-TSI < 110 ms [34]. These findings represented the first evaluation of a CMR measure of dyssynchrony against hard clinical endpoints in patients undergoing CRT. So as not to fall into the same problems as echocardiographic measures, [23-25] however, the CMR-TSI requires external validation before it is adopted in clinical practice.

**Figure 5:**
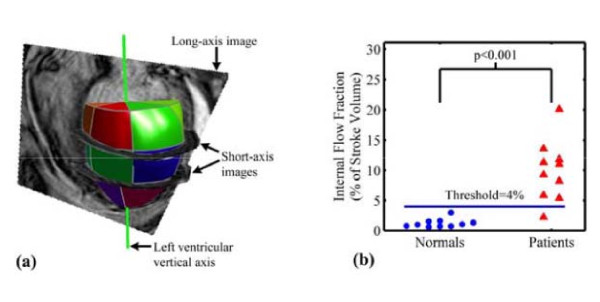
**Derivation of the internal flow fraction**. (a) For the calculation of internal flow fraction, the three-dimensional LV volume is superimposed on the four-chamber long-axis and short axis SSFP images and the left ventricle is divided into 16 wedge-shaped regional volumes. (b) the Internal Flow Fraction discriminates between patients and healthy controls with 95% accuracy. Reproduced with permission from Fornwalt BK, et al. [46]

Cine SSFP CMR has also been used to derive volumetric measures of dyssynchrony. In this regard, Forwalt et al have employed internal flow (IF) as a measure of the 'sloshing' of blood within the LV, reflecting the wasted work attributable to dyssynchrony. Using this technique, the LV volume is reconstructed and divided into wedge-shaped volumes. (Figure 5) Internal flow is defined as the sum of the magnitude of the volume changes in these regions minus the magnitude of the global volume change over each time step in the cardiac cycle: *IF*(*t*) = ∑|Δ*V*(*t*)_*regional*_| - |Δ∑*V*(*t*)_*regional*_|. Accordingly, this difference is zero if no IF has occurred. The IF fraction (IFF) is defined as the total internal flow as a percentage of stroke volume. Using this technique, Fornwalt et al found an IFF of 10 ± 5% in typical CRT patients (NYHA class III or IV, LVEF < 35%, QRS > 150 ms) and of 1 ± 1% in the healthy controls (p < 0.001) [46]. An IFF cut-off of 4% discriminated between patients and controls with 90% sensitivity and 100% specificity. Again, this SSFP technique is superior to echocardiographic measures of dyssynchrony in discriminating between patients with heart failure and healthy control subjects. Whether or not the IFF predicts the response and outcome of CRT remains to be explored.

#### CMR tagging

To accurately assess myocardial displacement and deformation (strain and strain rate), the imaging region of interest must be tracked through the cardiac cycle. In CMR, tacking can be achieved using myocardial tagging techniques which, in effect, label areas of myocardium. Essentially, tags, which are created by manipulating magnetization. [47], act as fiducial markers that conform to the myocardium in which they are placed. This not only permits accurate quantification of myocardial displacement, but also of strain and strain rate [48]. Commonly used sequences include spatial modulation of magnetization (SPAMM) and complimentary spatial modulation of magnetization (CSPAMM). The latter is more time-consuming, but it allows tags to be analysed in diastole as well as in systole.

**Figure 6:**
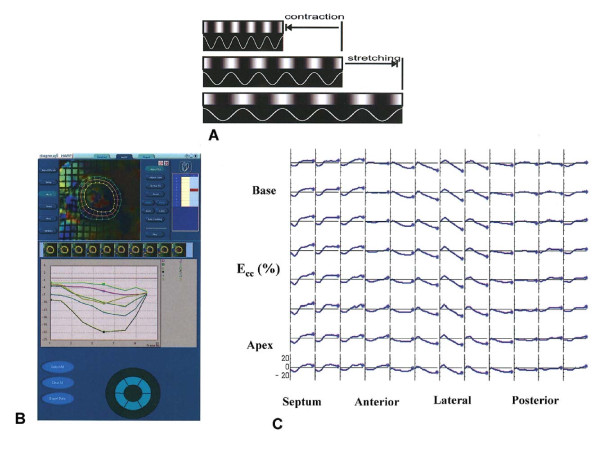
**Relationship between strain and tag frequency for harmonic phase-based strain measurements**. A)Contraction of a tagged fibre in the middle increases the tagging frequency (density of tag lines), as shown for the top fibre. Stretching causes a reduction in local frequency. (B) Two-dimensional segmentation of the heart and mesh generation (top panel) and regional strain versus time plots over the cardiac cycle; (C) complete regional strain versus time plots over the cardiac cycle for all segments. ECC = circumferential strain. Reproduced with permission from Lardo et al. [129]

A variety of techniques are available for the analysis of tagged images [48]. Harmonic phase (HARP, Diagnosoft, Inc., Palo Alto, California) analysis has recently gained popularity. This technique involves decomposition into harmonic magnitude and harmonic phase, which relate to cardiac structure and tag deformation, respectively. As the myocardium contracts and the tags get closer, the tag frequency increases. (Figure 6 and Additional File 2; **Movie 2**) This technique has been validated for the assessment of regional wall motion in animals and humans[49]. So far, however, HARP-derived indices of dyssynchrony have not been validated against clinical outcome measures after CRT.

**Figure 7:**
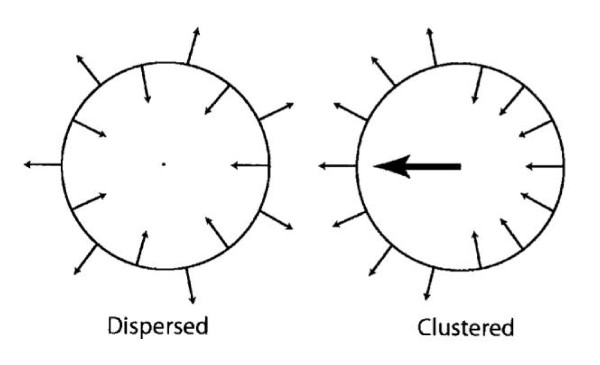
**Dyssynchrony indexes and regional distribution of wall motion**. Schematic representation of net dyssynchrony based on spatial distribution of delayed activation. When regions of delayed activation are clustered, the net impact on dysfunction is greater than when they are dispersed. Both situations are associated with similar overall numbers of delayed segments overall, as well as similar variance and dyssynchrony indexes. In contrast, a vector index would be zero for dispersed dyscoordinate shortening but non-zero when dyscoordinate motion is spatially clustered. Reproduced from Helm RH, et al. [52]

**Figure 8:**
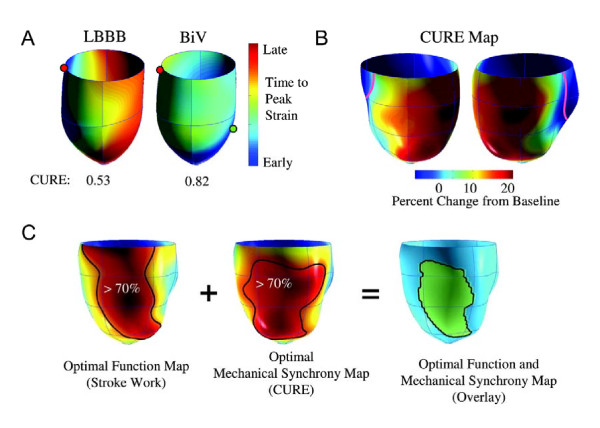
**Concordance between the CURE index and mechanical resynchronization**. A) Three-dimensional plot of relative mechanical activation time (time from QRS to peak circumferential strain) in a dog model of a dyssynchronous failing heart (with left bundle-branch block) and during CRT (biventricular pacing). The green dot shows the LV stimulation site. B) Synchrony indexed by CURE was calculated as a function of varying LV pacing site and plotted on three-dimensional maps, in which the colour red denotes optimal mechanical resynchronization. C) Combined maps derived for ventricular stroke work and synchrony (CURE) were determined in four failing hearts, and the territories producing optimal responses (70% maximal) for both were calculated and are displayed in green (far right). Adapted from Helm RH, et al. [54]

Most dyssynchrony measures reflect the temporal dispersion of cardiac events, measured in terms of the absolute difference or the standard deviation of the time to onset or peak wall motion, or deformation. In essence, they reflect temporal dispersion of these parameters. In addition, the number, or density of segments with late wall motion or deformation is likely to influence the hemodynamic effect of dyssynchrony. The so-called index of regional variance of strain, which is the percent of regional segments with delayed shortening, [50] represents a measure of 'density' of dyssynchrony. Importantly, however, this index does not take into account that for a given magnitude of such an index, wall motion or deformation can be clustered or evenly distributed. (Figure 7) In an attempt to circumvent this problem, Wyman et al used a regional variance vector of principal strain, according to which a unit vector sum only has a significant magnitude if delayed versus early regions are regionally clustered [51]. An alternative index is the temporal uniformity of strain, [52,53] otherwise known as the circumferential uniformity ratio estimate (CURE). The methodology used to derive the CURE involves generating time plots of strain for each segment in each short axis slice. Each plot of strain versus myocardial location is submitted to Fourier analysis to yield zero and first order terms. Using the CURE index and an animal model of tachy-pacing induced heart failure, Helm et al showed a spatial concordance between areas of maximal mechanical synchronization and areas of optimal LV systolic and diastolic performance after CRT (Figure 8) [54]. Bilchick et al have recently shown that a CURE cut-off of < 0.75 (with 0 denoting pure dyssynchrony and 1 denoting perfect synchrony) predicted improved NYHA class post-CRT with 90% accuracy (positive and predictive values: 87%, 100%) [55]. Whilst these findings are encouraging, multicentre studies are needed to address whether CURE not only predicts symptomatic response but also, morbidity and mortality.

#### Three-dimensional motion and strain

**Figure 9:**
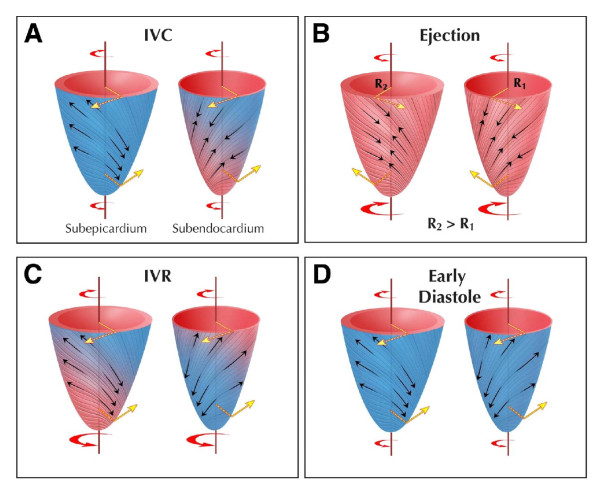
**Twist mechanics of the left ventricle**. Following electrical and mechanical activation in the apical subendocardial region, there follows a period of left ventricular isovolumic contraction (IVC), during which (A), the subendocardial myofibers (right-handed helix) shorten with stretching of the subepicardial myofibres (left-handed helix) to effect clockwise rotation of the apex and counterclockwise rotation of the base. During ejection (B), there is simultaneous shortening of the subendocardial and subepicardial layers. The larger arm of moment of the subepicardial fibers dominates the direction of twist, causing counterclockwise and clockwise rotation of the apex and base, respectively. During isovolumic relaxation (IVR) (C), subepicardial fibres lengthen from base to apex and subendocardial fibres lengthen from apex to base. In diastole, there is relaxation in both layers, with minimum untwisting (D). Reproduced with permission from Sengupta PP, et al. [56]

In conventional CMR tagging, tags in the form of an orthogonal grid are applied in the imaging plane. Motion analysis is therefore limited to two dimensions. The LV wall, however, is a complex structure in which myofibres are arranged in the form of a right-handed helix in the subendocardium and a left-handed helix in the subepicardium, with the mid-wall consisting of circumferential fibres. Such an architecture allows myocardial deformation in multiple planes (Figure 9) [56]. In LV systole, there is apical counterclockwise rotation and basal clockwise rotation around the LV long axis. In diastole, there is untwisting of the subendocardial layers, which contributes to diastolic suction. Simultaneously, the LV shortens in systole and lengthens in diastole. A dyssynchrony measure based on only one direction of motion or deformation may not necessarily reflect the extent of LV mechanical dysfunction.

**Figure 10:**
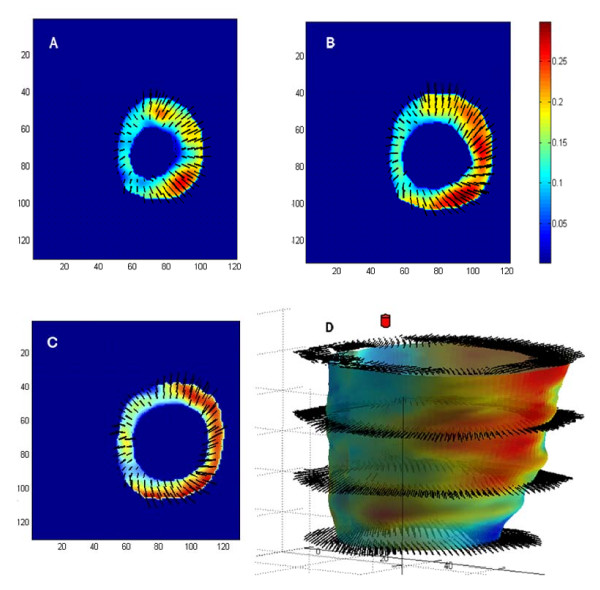
**Three-dimensional tagging and optical flow methodology**. Images from a technique involving a three-dimensional CMR tagging sequence and an optical flow method to measure three-dimensional LV wall deformation in a single cine acquisition. Panels A to C illustrate the maximum principal strain (***ε***_1_) on three representative short axis slices. The colour of the plot and the length of the overlaid line segments correspond to the magnitude of the strain while the direction is indicated by the vector. The images show the minimum ***ε***_1 _in the septum and the maximum ***ε***_1 _in the lateral wall. Panel D shows a mid-wall surface colour-coded by with ***ε***_1_, with superimposed eigenvector direction at four longitudinal levels. Note that ***ε***_1 _is directed towards the centre of the LV, indicating in-plane radial thickening.

Xu et al have recently developed a three-dimensional CMR tagging sequence and an optical flow method to measure three-dimensional LV wall deformation in a single cine acquisition [57]. As shown in Figure 10, this technique allows quantification of strain as well as direction of myocardial deformation. Although it has only been applied in animals, it is likely to prove useful in the field of CRT.

#### Other techniques

Various CMR techniques can be used to assess dyssynchrony from myocardial velocity measurements. In this respect, we should note the limitations of velocity-based measurements in the assessment of dyssynchrony, as demonstrated by echocardiographic studies [23,58].

Velocity-encoded CMR has shown some promise in the assessment of dyssynchrony [59]. In a small study, Marsan et al found a strong correlation between tissue Doppler imaging and velocity-encoded CMR with respect to longitudinal myocardial peak systolic and diastolic velocities and time to peak systolic velocity at the level of left ventricular septum and lateral walls. (r = 0.97, p < 0.001) [60] Similar findings emerged from a study of patients with idiopathic dilated cardiomyopathy [61].

Strain-encoded (SENC) CMR has recently emerged as a technique for directly imaging strain without the need for post-processing [62]. It uses a standard tagging sequence that tags myocardium at end-diastole with a sinusoidal tag pattern which modulates the longitudinal magnetization orthogonal to the imaging plane. Myocardial deformation leads to changes in the local frequency of the tag pattern in proportion to strain. As well as providing real-time quantitative strain measures, SENC has a higher spatial resolution than standard tagging and allows acquisition of both circumferential and longitudinal myocardial strain data. So far, however, this promising technique has not been applied to CRT.

#### Atrioventricular and interventricular dyssynchrony

The above CMR techniques are useful for the assessment of intraventricular dyssynchrony. Interventricular dyssynchrony, however, is also relevant in CRT. In a study of 45 patients undergoing CRT, Muellerleile et al found that the interventricular mechanical delay, derived from velocity-encoded CMR, was comparable to pulsed-wave echocardiography in predicting responders to CRT [63].

The role of atrioventricular dyssynchrony in predicting the response to CRT has not been studied. Arguably, correction of atrioventricular dyssynchrony should relate to a favourable response. Whilst atrioventricular dyssynchrony is easily measured with echocardiography, this is still challenging for CMR.

#### Dyssynchrony and LV pacing site

Several studies have shown that the anatomical position of the LV lead during CRT, assessed using fluoroscopy, has no bearing on the response to and outcome of CRT [64-66]. Small echocardiographic studies using tissue Doppler imaging, [67] tissue synchronization imaging (TSI), [68] three-dimensional echocardiography, [69] and speckle tracking, [70] however, have shown that a better response to CRT can be achieved if the LV lead is deployed in the area of latest activation or contraction. There is, nevertheless, a wide interindividual variation with respect to the site of latest activation [67-70].

Using the combination of pressure-volume loops and myocardial tagging to assess the ideal LV position in pacing-induced failing canine hearts, Helm et al found that LV sites yielding 70% of the maximal *d*P/*d*t_max _increase covered approx. 43% of the LV free wall [54]. The distribution and size of these pacing sites correlated with the three-dimensional dyssynchrony mapping derived from myocardial tagging. Essentially, this study provided proof of concept that myocardial tagging can be used to locate the ideal site for LV lead deployment in CRT. Importantly, however, this animal model did not involve myocardial infarction. Rademakers et al, on the other hand, have recently devised an animal model of heart failure involving myocardial infarction [71]. This model revealed that CRT can improve resynchronization and LV pump function to a similar degree in infarcted than non-infarcted hearts, but optimal lead positioning and timing of LV stimulation was crucial. In clinical practice, it is likely that combining three-dimensional myocardial tagging with an assessment of scar will be useful in guiding LV lead deployment.

#### Dyssynchrony mapping

If deploying the LV lead in an area of late activation or contraction is indeed important, it is in the interest of the CRT implanter to know how many areas there are and where they are. In this respect, we should consider that the myocardium is a complex anisotropic fibre structure, consisting of longitudinal, circumferential and oblique layers that form a mechanical link between remote areas of the myocardium [72-74]. The myocardium is also electrically heterogeneous from endocardium to mid-myocardium and epicardium [75]. Conduction disturbances, superimposed on the inherent anatomical, functional and electrical heterogeneity of the myocardium is likely to lead to multiple areas of dyssynchrony [76,77].

**Figure 11:**
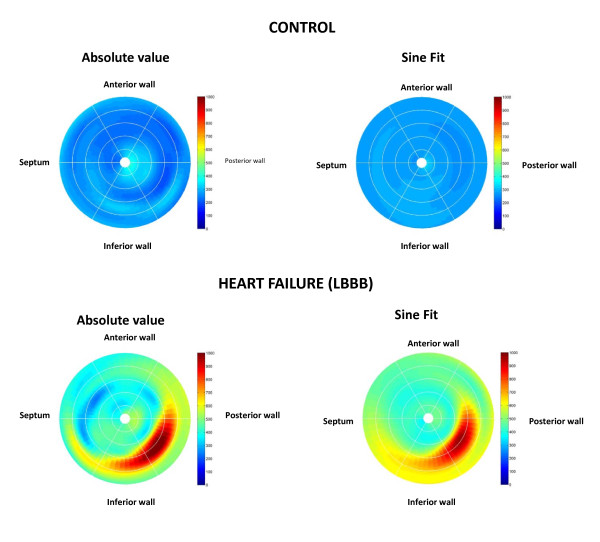
**Radial wall motion mapping with CMR**. The phase of inward radial wall motion is represented by colours ranging from blue (zero phase shift: inward motion during global systole), to green (90° phase shift: inward motion at the end of global systole) and to red (180° phase shift: inward motion during global diastole). Accordingly, the bull's eye with a homogenous blue colour throughout denotes complete synchrony, whereas a bull's eye with a homogenous red colour throughout denotes complete synchrony. Heterogenouss colour coding denotes dyssynchrony of radial motion, with blue representing early (global systolic phase) activation and red representing late (global diastolic phase) inward radial wall motion. Note the patchy distribution of wall motion throughout the LV. Reproduced with permission from Foley P, et al. [38]

Using in-house software written in MatLab (MatLab, The Mathworks Inc, MA; freely available from the author), we have used the phase of inward radial wall motion data derived from short axis SSFP slices to build colour-coded maps of radial wall motion (Figure 11) [38]. The spatial distribution of late inward radial motion was quantified by manually counting the number of patches with 180° phase shifts. We found that, in patients with ischemic cardiomyopathy, late inward radial motion is distributed in a patchy manner throughout the LV (Additional Files 3 and 4; **Movies 3 and 4**). This finding raises the possibility that deploying an LV lead over a single site of late wall motion may not correct global cardiac dyssynchrony. By the same token, multiple LV leads may be preferable to one LV lead in some patients, eg. non-responders to one LV lead [78]. Admittedly, this is speculative but may nevertheless be relevant to LV lead deployment.

### Myocardial scar and CRT

The effectiveness of almost all cardiac therapies is dependent on myocardial viability. Such is certainly the case for revascularization [79-84] and pharmacological therapy [85]. With respect to CRT, the total amount of scar (scar burden), its location and relationship to the pacing stimulus have been shown to be important in determining response and outcome. It is by virtue of unique anatomical resolution and the contrast between scarred and non-scarred myocardium achievable[81,86,87] that CMR has become the gold-standard for the *in vivo *assessment of myocardial scarring.

#### Scar burden

Several studies have shown that scar burden relates to a poor response to and outcome from CRT. In a LGE-CMR study of 28 patients undergoing CRT, White et al found that scar burden was higher in the non-responders versus responders group (median 24.7% vs. 1.0%, respectively, p = 0.0022) [88]. In a study of 45 patients with ischemic cardiomyopathy, we showed that scar burden correlated negatively with changes in 6-min walking distance (r = -0.54, p < 0.0001) and positively with changes in quality of life scores (r = 0.35, p = 0.028; high scores denote a poorer quality of life). The response (defined as survival for one year following implantation free of hospitalizations plus an improvement by ≥ 1 NYHA classes or by ≥ 25% in 6-min walking distance) in patients with < 33% scar was 2.3 times greater than in patients with ≥ 33% scar [89]. In a further study of 62 patients undergoing CRT, we found that the presence of a LV free wall scar was as an independent predictor of the composite endpoints of cardiovascular death or hospitalization for worsening heart failure [HR: 3.06, P < 0.0001)] as well as the endpoint of cardiovascular death [HR: 2.63, P = 0.0016)] after a mean follow-up period of 2 years. These findings are in keeping with those of a study using ^201^Tl single photon emission computed tomography (SPECT), [90] in which scar burden was shown to correlate negatively with changes in LVEF after CRT (r = -0.53; P < 0.0001).

The cut-off of scar burden above which CRT becomes ineffective is difficult to identify from the various studies. This is partly due to the adoption of different criteria for response to CRT and the inclusion of varying proportion of patients with ischemic and non-ischemic cardiomyopathy. For example, in our study of only patients with ischemic cardiomyopathy, [89] a scar volume of 33% was the best cut-off for predicting a favourable response: the responder rate in patients with < 33% scar was 2.3 times greater than patients with ≥ 33% scar. In contrast, White et al, who studies patients with ischemic (52%) and non-ischemic cardiomyopathy, found a scar burden < 15% as the best cut-off for predicting a clinical response [88]. Taken together, these studies of myocardial scarring and CRT support the hypothesis that there is a limit of scar burden above which resynchronization becomes ineffective.

#### Scar transmurality

**Figure 12:**
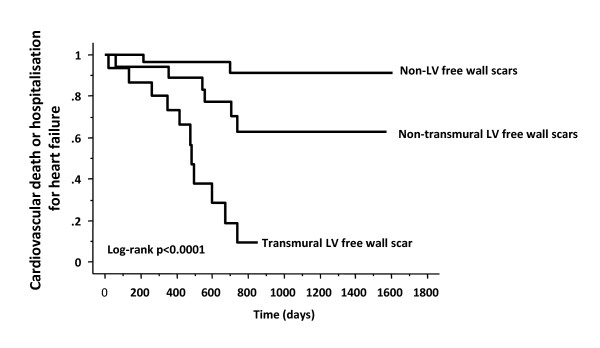
**Effect of scar transmurality on outcome after CRT**. Kaplan-Meier estimates of the time to clinical endpoints in patients with non-LV free wall scars, transmural LV free wall scars and non-transmural LV free wall scars. Adapted from Chalil S, et al. [89]

In the field of revascularization, myocardial segments with transmural scar respond poorly to revascularization [81]. We used LGE-CMR to assess the clinical effects of increasing transmurality of myocardial scars in LV free wall, the site of LV lead deployment in CRT. In a study patients with ischemic cardiomyopathy, a transmurality exceeding ≥ 51% in a LV free wall scar was associated with a poor response rate (23%), compared with scars with < 51% transmurality (88%, p < 0.001), in terms of a composite clinical score (survival for 1 year with no heart failure hospitalizations, and; improvement by ≥ 1 NYHA classes or ≥ 25% 6-min walking distance). In multivariate analyses, transmurality of LV free wall scars emerged as a negative predictor of clinical outcome after CRT. (Figure 12) [89].

#### Scar location and relationship to LV pacing site

**Figure 13:**
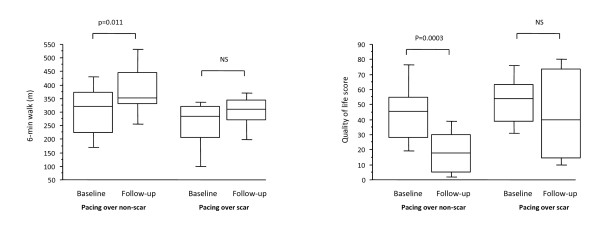
**Effect of LV pacing site scar on the clinical response to CRT**. Box and whisker plots of changes in 6-min walking distance and quality of life (QoL) in patients with LV free wall scars, grouped according to relationship of LV pacing lead tip to scar or non-scar. The five horizontal lines represent the 10^th^, 25^th^, 50^th^, 75^th ^and 90^th ^percentiles of each variable, from bottom to top. Reproduced from Chalil S, et al.[89]

**Figure 14:**
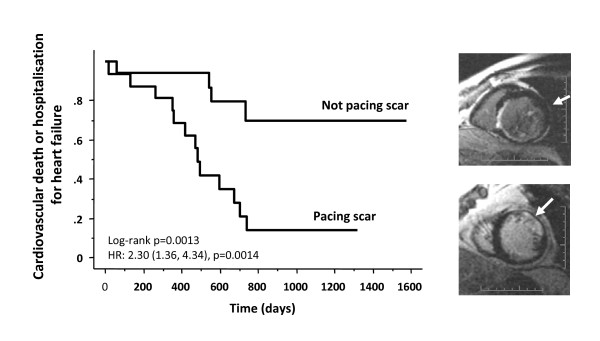
**Effect on clinical outcome of pacing LV free wall scar in CRT**. Kaplan-Meier estimates of the time to cardiovascular death or hospitalization for heart failure in patients with a LV free wall scar, grouped according to whether the LV lead was deployed over the scar or outside the scar. Hazard ratios and 95% confidence intervals derived from univariate Cox proportional hazards analyses are shown in parentheses. The right sided panels show representative short axis LGE-CMR slices of lead deployments on the scar and outside the scar. Adapted from Chalil S, et al. [95]

Intuitively, viability the paced LV segment could influence the outcome of CRT. This notion is supported by the fact that a pacing stimulus in scarred myocardium leads to a prolonged and fragmented QRS complex [91,92] as well as electrical and mechanical dyssynchrony. Furthermore, it is known that myocardial scars are not readily excitable [93] and that they effectively reduce the volume of myocardium available to a LV pacing stimulus [94]. In line with these findings, we have shown that pacing outside the LV free wall scar is associated with a better response than pacing over the scar (86% vs 33%, p = 0.004) (Figure 13). Another study showed that pacing scar was associated with a higher risk scar of cardiovascular death or heart failure hospitalization [81% vs 24%, p = 0.0009)], compared with pacing non-scar. (Figure 14)[95] These findings are in keeping with those of a study involving ^201^Tl single photon emission computed tomography, in which scar density in the segments in the vicinity of the LV lead was lower in responders versus non-responders (response defined as a ≥ 15% increase in LVEF) [90].

Following the finding that pacing LV free wall scar is detrimental in CRT is the use of LGE-CMR to guide LV lead deployment has become standard practice in some centres. In the author's implanting experience, scarring over the entire LV free wall is a rare occurrence and it is unusual not to have coronary sinus tributaries over non-scarred myocardium.

### Composite predictors

Heart failure is a complex syndrome that can hardly be quantified in terms of a single parameter. Accordingly, it is perhaps folly to consider that one single parameter can be used to predict the response and outcome of CRT. Clearly, the outcome of heart failure and CRT are intimately dependent on a panoply of factors, not all of which relate to hemodynamic or imaging parameters. It is this which provides the biological rationale for using composite predictors. The statistical attraction is that composite predictor tends to dampen the background noise of sampling error [96].

**Figure 15:**
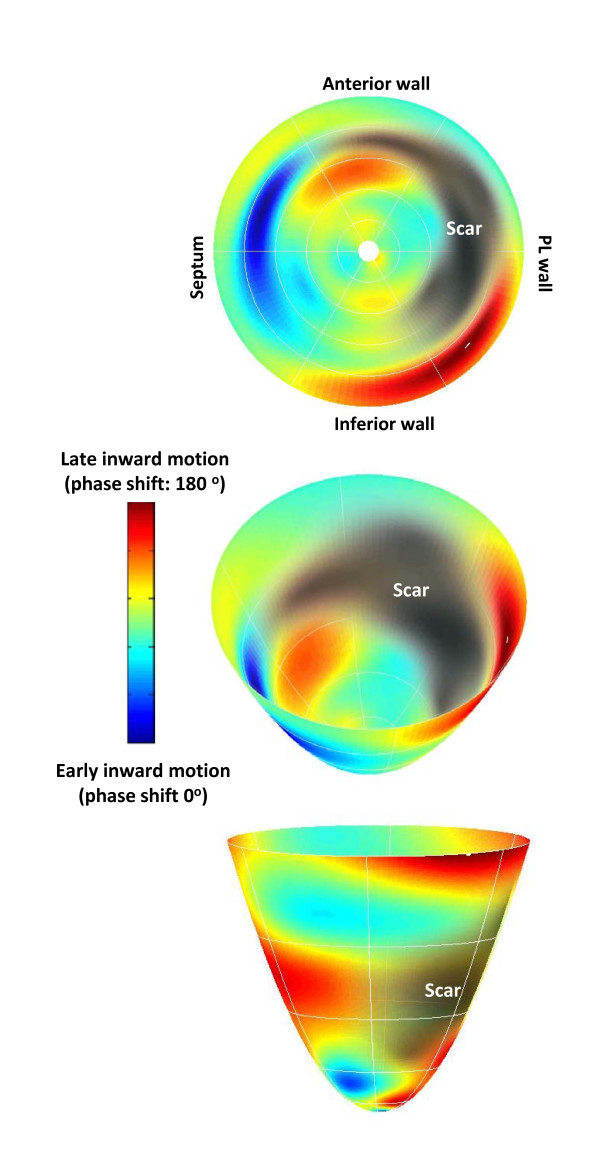
**Combined dyssynchrony and scar maps**. Endocardial wall motion and scar were quantified using SSFP and LGE-CMR, respectively. The color-encoded regional delay of radial inward motion is mapped onto a bull's eye map and LV model, pictured from above (middle figure) and below (bottom figure). Timing of radial inward motion is expressed as a phase delay ranging from zero to 180°. A phase delay of zero represents early ventricular motion concordant with initial electrical activation and is colour coded blue, while a phase delay of 180° denotes diastolic inward motion and is colour coded red. The LV free wall scar (grey-black) is superimposed on the endocardial wall motion map. In the figure, the LV septum is contracts early in systole and the inferior wall close to the LV free wall scar shows abnormal diastolic radial inward motion. Note the patchy distribution of wall motion throughout the left ventricle. Reproduced with permission from Foley P, et al. [38]

**Figure 16:**
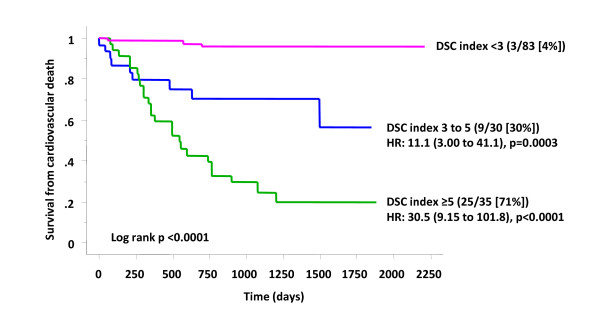
**The DSC index as a composite predictor of outcome after CRT**. Graph shows Kaplan-Meier estimates of the time to cardiovascular death. Patients were stratified according to pre-implant Dyssynchrony, left ventricular free wall Scar and Creatinine (DSC) index. The event rate, number of patients in the DSC risk stratum and the% event rate are shown in parentheses. The hazard ratio (HR) and 95% confidence intervals are also shown. Reproduced with permission from Leyva F, et al. [130]

In patients not treated with CRT, composite predictors have been shown to be superior to single parameters in prognostic risk assessment [97-99]. We have recently evaluated 16 risk factors in relation to mortality and morbidity in 148 patients undergoing CRT [100]. These risk factors included the CMR-TSI scar location, derived from SSFP and LGE-CMR, respectively. (Figure 15) In Cox proportional hazards analyses, CMR-derived Dyssynchrony, LV Scar location and Creatinine (the DSC index) emerged as independent predictors of cardiovascular mortality. The DSC index, derived from these variables combined, emerged as a powerful predictor of cardiovascular mortality. Compared with patients with a DSC < 3, cardiovascular mortality in patients in the intermediate (DSC index: 3 to 5; HR: 11.1) and high (DSC index ≥ 5; HR: 30.5) were higher (Figure 16). These findings illustrate that combining known predictors of cardiovascular death in patients with heart failure, such as creatinine, [98,101-104] with variables which are known to be pathophysiologically linked to CRT, such as dyssychrony and LV scar, improves the ability to predict outcome. Other groups have also used combined measures to predict the response to CRT. Bilchick et al, for example, found that adding LGE-CMR scar data (< 15% scar) to a dyssynchrony measure (in the form of the CURE index) improved the ability to predict an improvement in NYHA class after CRT [55].

### CMR and etiology of heart failure

Heart failure is not a diagnosis without qualification of etiology [105]. The etiology of heart failure influences prognosis and the choice of therapy, including device therapy. In this respect, the 2007 UK National Institute of Clinical Excellence (NICE) guidance stipulated that CRT-D should only be considered if there is a history of a myocardial infarction, or 'a familial cardiac condition with a history of sudden death, including long QT syndrome, hypertrophic cardiomyopathy, Brugada syndrome or arrhythmogenic right ventricular dysplasia, or have undergone surgical repair of congenital heart disease' [44]. Idiopathic dilated cardiomyopathy, which accounts for most cases of non-ischemic cardiomyopathy, was not considered by NICE and therefore, falls under the guidance for CRT-P. In the UK, therefore, etiology is particularly important in choosing between CRT-P or CRT-D. Importantly, however, an ischemic etiology does not imply reduced prognostic benefit from CRT [106].

The diagnosis of ischemic cardiomyopathy has traditionally been made on the basis of a history of a myocardial infarction, [107] the finding of coronary artery stenoses on coronary angiography or of a regional wall motion abnormalities on echocardiography. It is well recognized, however, that myocardial infarctions can be silent (28% in men, 35% in women), [108] that coronary angiography can be normal after a myocardial infarction (8%) [109,110] and that wall motion abnormalities are not exclusive to ischemic cardiomyopathy [111].

**Figure 17:**
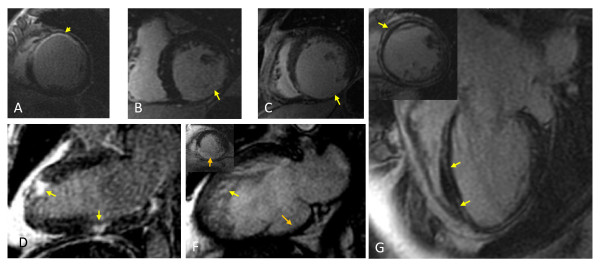
**Assessment of heart failure etiology with CMR**. A) LGE-CMR short axis slice showing a subendocardial myocardial infarction in the territory of the left anterior descending artery; B) LGE-CMR short axis slice showing a transmural myocardial infarction the territory of the circumflex artery; C) LGE-CMR showing a transmural myocardial infarction in the territory of the right coronary artery, which is associated with marked myocardial thinning.; D) Patchy LGE characteristic of myocarditis.; F) Mixed cardiomyopathy: left ventricular non-compaction cardiomyopathy and ischemic cardiomyopathy. Note the transmural inferior myocardial infarction (see insert for LGE-CMR) which has led to myocardial thinning.; G) Four-chamber and short-axis LGE-CMR images showing mid-wall LGE, denoting fibrosis, in idiopathic dilated cardiomyopathy.

Unparalelled anatomical imaging, coupled with the findings of late gadolinium enhancement (LGE)-CMR, [112,113] makes CMR an ideal, radiation-free investigation for the investigation of heart failure etiology. (Figure 17) Typically, a myocardial infarction leads to scarring in subendocardial or transmural distribution along arterial territories. In contrast, non-ischemic cardiomyopathy is characterized by a lack of localized myocardial scarring or less often, by mid-wall LGE, denoting fibrosis [86,87,114]. A patchy distribution of LGE is found in myocarditis, sarcoidosis and arrhythmogenic right ventricular cardiomyopathy. Diffuse LGE is characterisitic of amyloidosis and Anderson-Fabry disease.

The sequences used to quantify T2* have proven to be unique in the identification and management of iron overload cardiomyopathy, [115] Sequences using T2-weighting have also been used to identify myocardial oedema, [116,117] which may be useful in the assessment of myocarditis and acute coronary syndromes [110,87].

### CMR device compatibility

As discussed above, the principal role of imaging in CRT is in selecting patients and in guiding device implantation. Potential aspects of imaging after device implantation, such as residual dyssynchrony and relationship of myocardial scarring to the implanted LV lead may help identifying the reasons for a lack of response.

Several factors preclude the use of CMR in patients with devices [118]. Radiofrequency-induced heating of the pacing leads has been shown to lead to temperatures as high as 60° in experimental settings [119]. On the other hand, radiofrequency and magnetic gradients induce currents within the device generator [120] and these can interfere with detection and pacing algorithms. In addition, they can induce arrhythmias and alter pacemaker settings. These factors have indeed implicated in the reported deaths. Despite these concerns, several centres have recently reported favourably on the safety of CMR in patients with pacemakers [121,122]. Continuous ECG, blood pressure and oxygen saturation monitoring during the scan, turning off the device in non device-dependent patients, setting the leads to a bipolar configuration and using low field, gradient and radiofrequency settings are among the precautionary measures taken. With regard to CRT-D and ICDs, experience is more limited. In balance, the strength of the evidence for a role of CMR in CRT is insufficient to justify scanning after implantation.

This year has seen the launch of the first CMR-safe pacemaker (Medtronic Inc, Minneapolis, US). The development of CMR-safe CRT-P and CRT-D devices would permit assessment of cardiac function, dyssynchorny and myocardial viability after device implantation. This is not only likely to help in clinical management but it will undoubtedly further our understanding of the mechanisms underlying CRT.

### Intraoperative CMR in CRT

Recently, Schwatzman have shown how myocardial scar, imaged pre-operatively using LGE-CMR, can be fused with electroanatomic mapping data to guide LV lead implantation [123]. Using this technique, activation time and virtual venography was used to target LV lead positions, and the electroanatomic mapping system was used to assist in lead manipulation. Further studies, however, are needed to determine whether other CMR-derived data, relating to coronary sinus anatomy, [124] mechanical activation, [125,34] perfusion and viability can also be 'fused' with real-time CMR or conventional fluoroscopy to guide LV lead deployment.

## Conclusions

This decade is likely to see an exponential growth in the use of CRT for patients with heart failure. CMR not only provides unparalleled quality of imaging for cardiac structure and function but it is also unique in differentiating between the various causes of LV dysfunction. It is on this basis that CMR is already considered as an ideal 'one-stop' investigation for patients with heart failure. In addition, perhaps the most clinically applicable aspect of CMR to CRT *per se *is its ability to precisely localise myocardial scar, which is known to be crucial in LV lead deployment. For these reasons, CMR has a clear role in the diagnostic and implantation pathway of patients undergoing CRT. Further studies are needed to clarify the utility of fusion imaging in guiding LV lead deployment. On the part of scanner manufacturers, further software development and validation is required, so as to make analysis of dyssynchrony, tagging and LGE data more accessible to the clinician. With the development of CMR-compatible devices, the use of CMR in CRT device optimization may one day become a reality.

## Conflict of interests

The authors declare that they have no competing interests.

## Supplementary Material

Additional file 1 **Movie 1**. Four-chamber cine SSFP in a patient with idiopathic dilated cardiomyopathy and a QRS of 98 ms, illustrating how visually-detectable dyssynchrony can also occur in patients with a narrow QRS complex. Click here for file

Additional file 2 **Movie 2**. Shows colour-coded analysis of myocardial tags using HARP. x-coordinate represents time frames, y-coordinate represents percent circumferential shortening (% Ecc). Reproduced with permission from Shehata ML, et al [48] Click here for file

Additional file 3 **Movie 3**. Colour-coded endocardial wall motion throughout the cardiac cycle in a healthy subject, derived from SSFP imaging. Note the homogeneity of colour (early wall motion in blue and late wall motion in red) throughout the cardiac cycle. Click here for file

Additional file 4 **Movie 4**. Colour-coded endocardial wall motion throughout the cardiac cycle in a patients with heart failure and a left bundle branch block, derived from SSFP imaging. Note the heterogeneity of colour (early wall motion in blue and late wall motion in red) throughout the cardiac cycle. Click here for file

## References

[B1] Cazeau S Ritter P Bachdach S Lazarus A Limousin M Henao L Mundler O Daubert JC Mugica J Four chamber pacing in dilated cardiomyopathy Pacing Clin Electrophysiol 1994 17 11 Pt 2 1974 1979 10.1111/j.1540-8159.1994.tb03783.x 7845801

[B2] Leclercq C Cazeau S Le Breton H Ritter P Mabo P Gras D Pavin D Lazarus A Daubert JC Acute hemodynamic effects of biventricular DDD pacing in patients with end stage heart failure J Am Coll Cardiol 1998 32 7 1825 1831 10.1016/S0735-1097(98)00492-6 9857858

[B3] Auricchio A Stellbrink C Block M Sack S Vogt J Bakker P Klein H Karmer A Ding J Salo R Effect of pacing chamber and atrioventricular delay on acute systolic function of paced patients with congestive heart failure Circulation 1999 99 2993 3001 10368116 10.1161/01.cir.99.23.2993

[B4] Cazeau S Leclercq C Lavergene T Walker S Varma C Linde C Garrigue S Kappenberger L Haywood GA Santini M Effects of multisite biventricular pacing in patients with heart failure and interventricular conduction delay N Engl J Med 2001 344 873 880 10.1056/NEJM200103223441202 11259720

[B5] Cleland JGF Daubert JC Erdmann E Freemantle N Gras D Kappenberger L Tavazzi L for the Cardiac Resynchronization-Heart Failure (CARE-HF) study investigators. The effect of cardiac resynchronization on morbidity and mortality in heart failure N Eng J Med 2005 352 1539 1549 10.1056/NEJMoa050496 15753115

[B6] Bristow MR Saxon LA Boehmer J Krueger S Kass D De Marco T Carson P DiCarlo L DeMets D White BG for the Comparison of Medical Therapy, Pacing and Defibrillation in Heart Failure (COMPANION) Investigators. Cardiac resynchronization therapy with or without an implantable defibrillator in advanced heart failure N Eng J Med 2004 350 2140 2150 10.1056/NEJMoa032423 15152059

[B7] Moss AJ Hall WJ Cannom DS Klein H Brown MW Daubert JP Estes NA Foster E Greenberg H Higgins SL Cardiac-resynchronization therapy for the prevention of heart-failure events N Engl J Med 2009 361 14 1329 1338 10.1056/NEJMoa0906431 19723701

[B8] Daubert C Gold MR Abraham WT Ghio S Hassager C Goode G Szili-Torok T Linde C Prevention of disease progression by cardiac resynchronization therapy in patients with asymptomatic or mildly symptomatic left ventricular dysfunction: insights from the European cohort of the REVERSE (Resynchronization Reverses Remodeling in Systolic Left Ventricular Dysfunction) trial J Am Coll Cardiol 2009 54 20 1837 1846 10.1016/j.jacc.2009.08.011 19800193

[B9] Funck RC Blanc JJ Mueller HH Schade-Brittinger C Bailleul C Maisch B Biventricular stimulation to prevent cardiac desynchronization: rationale, design, and endpoints of the 'Biventricular Pacing for Atrioventricular Block to Prevent Cardiac Desynchronization (BioPace)' study Europace 2006 8 8 629 635 10.1093/europace/eul075 16864616

[B10] Holzmeister J Hurlimann D Steffel J Ruschitzka F Cardiac resynchronization therapy in patients with a narrow QRS Current heart failure reports 2009 6 1 49 56 10.1007/s11897-009-0009-5 19265593

[B11] Foley PW Leyva F Frenneaux MP What is treatment success in cardiac resynchronization therapy? Europace 2009 11 Suppl 5 v58 65 10.1093/europace/eup308 19861392 PMC2768584

[B12] Effects of enalapril on mortality in severe congestive heart failure. Results of the Cooperative North Scandinavian Enalapril Survival Study (CONSENSUS). The CONSENSUS Trial Study Group N Engl J Med 1987 316 23 1429 1435 10.1056/NEJM198706043162301 2883575

[B13] A randomized trial of beta-blockade in heart failure. The Cardiac Insufficiency Bisoprolol Study (CIBIS). CIBIS Investigators and Committees Circulation 1994 90 4 1765 1773 7923660 10.1161/01.cir.90.4.1765

[B14] Pitt B Zannad F Remme WJ for the Randomized Aldactone Evaluation Study Investigators The effect of spironolactone on morbidity and mortality in patients with severe heart failure N Engl J Med 1999 341 709 717 10.1056/NEJM199909023411001 10471456

[B15] Prinzen FW Augustijn CH Arts T Allessie MA Reneman RS Redistribution of myocardial fiber strain and blood flow by asynchronous activation Am J Physiol 1990 259 H300 H308 2386214 10.1152/ajpheart.1990.259.2.H300

[B16] Park RC Little WC O'Rourke RA Effect of alteration of left ventricular activation sequence on the left ventricular end-systolic pressure-volume relation in closed-chest dogs Circ Res 1985 57 5 706 717 4053304 10.1161/01.res.57.5.706

[B17] Burkhoff D Oikawa RY Sagawa K Influence of pacing site on canine left ventricular contraction Am J Physiol 1986 251 H428 H435 3740295 10.1152/ajpheart.1986.251.2.H428

[B18] Pitzalis MV Iacoviello M Romito R Guida P De Tomasi E Luzzi G Anaclerio M Forleo C Rizzon P Cardiac resynchronization therapy tailored by echocardiographic evaluation of ventricular asynchrony J Am Col Cardiol 2002 40 1615 1622 10.1016/S0735-1097(02)02337-9 12427414

[B19] Yu CM Zhang Q Chan YS Chan CK Yip GW Kum LC Wu EB Lee PW Lam YY Chan S Tissue Doppler velocity is superior to displacement and strain mapping in predicting left ventricular reverse remodelling response after cardiac resynchronisation therapy Heart 2006 92 10 1452 1456 10.1136/hrt.2005.083592 16621873 PMC1861066

[B20] Yu CM Fung WH Lin H Zhang Q Sanderson JE Lau CP Predictors of left ventricular reverse remodeling after cardiac resynchronization therapy for heart failure secondary to idiopathic dilated or ischemic cardiomyopathy Am J Cardiol 2003 91 6 684 688 10.1016/S0002-9149(02)03404-5 12633798

[B21] Bax JJ Bleeker GB Marwick TH Molhoek SG Boersma E Steendijk P Van der Wall EE Schalij MJ Left ventricular dyssynchrony predicts response and prognosis after cardiac resynchronization therapy J Am Col Cardiol 2004 44 1834 1840 10.1016/j.jacc.2004.08.016 15519016

[B22] Chung E Leon A Tavazzi L Sun J Nihoyannopoulos P Merlino J Abraham W Guio S Leclerq C Bax J Results of the Predictors of Response to CRT (PROSPECT) Trial Circulation 2008 117 20 2608 2616 10.1161/CIRCULATIONAHA.107.743120 18458170

[B23] Hawkins NM Petrie MC Burgess MI McMurray JJ Selecting patients for cardiac resynchronization therapy: the fallacy of echocardiographic dyssynchrony J Am Coll Cardiol 2009 53 21 1944 1959 10.1016/j.jacc.2008.11.062 19460607

[B24] Marwick T Hype and hope in the use of echocardiography for selection for cardiac resynchronization therapy: the tower of Babel revisited Circulation 2008 117 2573 2576 10.1161/CIRCULATIONAHA.108.772475 18490533

[B25] Conca C Faletra FF Miyazaki C Oh J Mantovani A Klersy C Sorgente A Pedrazzini GB Pasotti E Moccetti T Echocardiographic parameters of mechanical synchrony in healthy individuals Am J Cardiol 2009 103 1 136 142 10.1016/j.amjcard.2008.08.039 19101244

[B26] Fornwalt BK Sprague WW BeDell P Suever JD Gerritse B Merlino JD Fyfe DA Leon AR Oshinski JN Agreement Is Poor Among Current Criteria Used to Define Response to Cardiac Resynchronization Therapy Circulation 2010 121 18 1985 1991 10.1161/CIRCULATIONAHA.109.910778 20421518 PMC2882855

[B27] Kass D An epidemic of dyssynchrony J Am Coll Cardiol 2008 51 12 17 10.1016/j.jacc.2007.09.027 18174030

[B28] Nagueh S Mechanical dyssynchrony in congestive heart failure J Am Coll Cardiol 2008 51 18 22 10.1016/j.jacc.2007.08.052 18174031

[B29] Yu CM Yang H Lau CP Wang Q Wang S Lam L Sanderson JE Regional left ventricle mechanical asynchrony in patients with heart disease and normal QRS duration: implication for biventricular pacing therapy Pacing Clin Electrophysiol 2003 26 2 Pt 1 562 570 10.1046/j.1460-9592.2003.00095.x 12710315

[B30] Yu CM Lin H Zhang Q Sanderson JE High prevalence of left ventricular systolic and diastolic asynchrony in patients with congestive heart failure and normal QRS duration Heart 2003 89 54 60 10.1136/heart.89.1.54 12482792 PMC1767510

[B31] Haghjoo M Bagherzadeh A Fazelifar A Haghighi Z Esmaielzadeh M Alizadeh A Emkanjoo Z Sadeghpour A Samiei N Farahani M Prevalence of mechanical dyssynchrony in heart failure patients with different QRS durations Pacing Clin Electrophysiol 2007 30 616 622 10.1111/j.1540-8159.2007.00722.x 17461871

[B32] Takemoto Y Hozumi T Sugioka K Takagi Y Matsumura Y Yoshiyama M Abraham T Yoshikawa J Beta-blocker therapy induces ventricular resynchronization in dilated cardiomyopathy with narrow QRS complex J Am Coll Cardiol 2007 49 778 783 10.1016/j.jacc.2006.05.081 17306707

[B33] Marcassa C Campini R Verna E Ceriani L Giannuzzi P Assessment of cardiac asynchrony by radionuclide phase analysis: correlation with ventricular function in patients with narrow or prolonged QRS interval Eur J Heart Fail 2007 9 484 490 10.1016/j.ejheart.2007.01.002 17347038

[B34] Chalil S Stegemann B Muhyaldeen S Khadjooi K Smith R Jordan P Leyva F Intraventricular Dyssynchrony Predicts Mortality and Morbidity Following Cardiac Resynchronization Therapy: A Study Using Cardiovascular Magnetic Resonance Tissue Synchronization Imaging J Am Coll Cardiol 2007 50 243 252 10.1016/j.jacc.2007.03.035 17631217

[B35] Turner M Bleasdale R Vinereanu D Mumford C Paul V Fraser A Frenneaux M Electrical and mechanical components of dyssynchrony in heart failure patients with normal QRS duration and left bundle-branch block: impact of left and biventricular pacing Circulation 2004 109 2544 2549 10.1161/01.CIR.0000131184.40893.40 15148267

[B36] Wang J Kurrelmeyer K Torre-Amione G Nagueh S Systolic and diastolic dyssynchrony in patents with diastolic heart failure and the effect of medical therapy J Am Coll Cardiol 2007 49 88 96 10.1016/j.jacc.2006.10.023 17207727

[B37] Chan CP Zhang Q Yip GW Fung JW Lam YY Lee PW Wu EB Shang Q Liang Y Yu CM Relation of left ventricular systolic dyssynchrony in patients with heart failure to left ventricular ejection fraction and to QRS duration Am J Cardiol 2008 102 5 602 605 10.1016/j.amjcard.2008.04.032 18721520

[B38] Foley PW Khadjooi K Ward JA Smith RE Stegemann B Frenneaux MP Leyva F Radial dyssynchrony assessed by cardiovascular magnetic resonance in relation to left ventricular function, myocardial scarring and QRS duration in patients with heart failure J Cardiovasc Magn Reson 2009 11 1 50 56 10.1186/1532-429X-11-50 19930713 PMC2789061

[B39] Achilli A Sassara M Ficili S Pontillo D Achilli P Alessi C De Spirito S Guerra R Patruno N Serra F Long-term effectiveness of cardiac resynchronization therapy in patients with refractory heart failure and "narrow" QRS J Am Coll Cardiol 2003 42 2117 2124 10.1016/j.jacc.2003.08.024 14680737

[B40] Yu CM Chan Y Zhang Q Yip G Chan C Kum L Wu L Lee A Lam Y Fung J Benefits of cardiac resynchronization therapy for heart failure patients with narrow QRS complexes and coexisting systolic asynchrony by echocardiography J Am Coll Cardiol 2006 48 2251 2257 10.1016/j.jacc.2006.07.054 17161255

[B41] Gasparini M Mantica M Galimberti P Marconi M Genovese L Faletra F Simonini S Klersy C Coates R Gronda E Beneficial effects of biventricular pacing in patients with a "narrow" QRS Pacing Clin Electrophysiol 2003 26 1 Pt 2 169 174 10.1046/j.1460-9592.2003.00010.x 12687806

[B42] Beshai J Grimm R Nagueh S Baker J Beau S Greenberg S Pires L Tchou P for the RethinQ Study Investigators Cardiac-resynchronization therapy in heart failure with narrow QRS complexes N Eng J Med 2007 357 2461 2471 10.1056/NEJMoa0706695 17986493

[B43] Auricchio A Leyva F Inclusion and exclusion criteria for CRT Heart Rhythm 2009 6 8 1235 1237 10.1016/j.hrthm.2009.03.053 19632640

[B44] Barnett D Phillips S Longson C Cardiac resynchronisation therapy for the treatment of heart failure: NICE technology appraisal guidance Heart 2007 93 1134 1135 10.1136/hrt.2007.127563 17699177 PMC1955017

[B45] Koos R Neizel M Schummers G Krombach GA Stanzel S Gunther RW Kelm M Kuhl HP Feasibility and initial experience of assessment of mechanical dyssynchrony using cardiovascular magnetic resonance and semi-automatic border detection J Cardiovasc Magn Reson 2008 10 1 49 10.1186/1532-429X-10-49 18983646 PMC2588580

[B46] Fornwalt BK Gonzales PC Delfino JG Eisner R Leon AR Oshinski JN Quantification of left ventricular internal flow from cardiac magnetic resonance images in patients with dyssynchronous heart failure J Magn Reson Imaging 2008 28 2 375 381 10.1002/jmri.21446 18666147

[B47] Axel L Dougherty L MR imaging of motion with spatial modulation of magnetization Radiology 1989 171 3 841 845 2717762 10.1148/radiology.171.3.2717762

[B48] Shehata ML Cheng S Osman NF Bluemke DA Lima JA Myocardial tissue tagging with cardiovascular magnetic resonance J Cardiovasc Magn Reson 2009 11 1 55 10.1186/1532-429X-11-55 20025732 PMC2809051

[B49] Kraitchman DL Sampath S Castillo E Derbyshire JA Boston RC Bluemke DA Gerber BL Prince JL Osman NF Quantitative ischemia detection during cardiac magnetic resonance stress testing by use of FastHARP Circulation 2003 107 15 2025 2030 10.1161/01.CIR.0000062684.47526.47 12668517

[B50] Nelson GS Curry CW Wyman BT Kramer A Declerck J Talbot M Douglas MR Berger RD McVeigh ER Kass DA Predictors of systolic augmentation from left ventricular pre excitation in patients with dilated cardiomyopathy and intraventricular conduction delay Circulation 2000 101 23 2703 2709 10851207 10.1161/01.cir.101.23.2703

[B51] Wyman BT Hunter WC Prinzen FW Faris OP McVeigh ER Effects of single-and biventricular pacing on temporal and spatial dynamics of ventricular contraction Am J Physiol Heart Circ Physiol 2002 282 H372 H379 11748084 10.1152/ajpheart.2002.282.1.H372

[B52] Helm RH Lecquercq C Faris Q Ozturk C McVeigh E Lardo AC Kass D Cardiac dyssynchrony analysis using circumferential versus longitudinal strain: Implications for assessing cardiac resynchronization Circulation 2005 111 2760 2767 10.1161/CIRCULATIONAHA.104.508457 15911694 PMC2396330

[B53] Leclercq C Fairs O Tunin R Johnson J Kato R Evans F Spinelli J Halperin H McVeigh E Kass DA Systolic improvement and mechanical resynchronization does not require electrical synchrony in the dilated failing heart with left bundle branch block Circulation 2002 106 1760 1763 10.1161/01.CIR.0000035037.11968.5C 12356626

[B54] Helm RH Byrne M Helm PA Daya SK Osman NF Tunin R Halperin HR Berger RD Kass DA Lardo AC Three-dimensional mapping of optimal left ventricular pacing site for cardiac resynchronization Circulation 2007 115 8 953 961 10.1161/CIRCULATIONAHA.106.643718 17296857

[B55] Bilchick KC Dimaano V Wu KC Helm RH Weiss RG Lima JA Berger RD Tomaselli GF Bluemke DA Halperin HR Cardiac magnetic resonance assessment of dyssynchrony and myocardial scar predicts function class improvement following cardiac resynchronization therapy Jacc 2008 1 5 561 568 19356481 10.1016/j.jcmg.2008.04.013 PMC2678755

[B56] Sengupta PP Khandheria BK Narula J Twist and untwist mechanics of the left ventricle Heart failure clinics 2008 4 3 315 324 10.1016/j.hfc.2008.03.001 18598983

[B57] Xu C Pilla J Isaac G Gorman J Blom A Gorman R Ling Z Dougherty L Deformation analysis of 3D tagged cardiac images using an optical flow method J Cardiovasc Magn Reson 2010 12 1 19 25 10.1186/1532-429X-12-19 20353600 PMC2856559

[B58] Miyazaki C Powell BD Bruce CJ Espinosa RE Redfield MM Miller FA Hayes DL Cha YM Oh JK Comparison of echocardiographic dyssynchrony assessment by tissue velocity and strain imaging in subjects with or without systolic dysfunction and with or without left bundle-branch block Circulation 2008 117 20 2617 2625 10.1161/CIRCULATIONAHA.107.733675 18474810

[B59] Constable RT Rath KM Sinusas AJ Gore JC Development and evaluation of tracking algorithms for cardiac wall motion analysis using phase velocity MR imaging Magn Reson Med 1994 32 1 33 42 10.1002/mrm.1910320106 8084235

[B60] Marsan NA Bleeker GB van Bommel RJ Ypenburg C Delgado V Borleffs CJ Holman ER van der Wall EE Schalij MJ Bax JJ Comparison of time course of response to cardiac resynchronization therapy in patients with ischemic versus nonischemic cardiomyopathy Am J Cardiol 2009 103 5 690 694 10.1016/j.amjcard.2008.11.008 19231335

[B61] Westenberg JJM Lamb H van der Geest RJ Bleeker GA Holman ER Schalij MJ de Roos A Van der Wall EE Reiber JHC Bax JJ Assessment of left ventricular dyssynchrony in patients with conduction delay and idiopathic dilated cardiomyopathy:head-to-head comparison between tissue Doppler imaging and velocity-encoded magnetic resonance imaging J Am Coll Cardiol 2006 47 2042 2048 10.1016/j.jacc.2006.01.058 16697323

[B62] Osman NF Sampath S Atalar E Prince JL Imaging longitudinal cardiac strain on short-axis images using strain-encoded MRI Magn Reson Med 2001 46 2 324 334 10.1002/mrm.1195 11477637

[B63] Muellerleile K Baholli L Groth M Barmeyer AA Koopmann K Ventura R Koester R Adam G Willems S Lund GK Interventricular mechanical dyssynchrony: quantification with velocity-encoded MR imaging Radiology 2009 253 2 364 371 10.1148/radiol.2532090145 19703849

[B64] Gasparini M Mantica M Galimberti P Bocciolone M Genovese L Mangiavacchi M Marchesina UL Faletra F Klersy C Coates R Is the left ventricular lateral wall the best lead implantation site for cardiac resynchronization therapy? Pacing Clin Electrophysiol 2003 26 1 Pt 2 162 168 10.1046/j.1460-9592.2003.00009.x 12687805

[B65] Rossillo A Verma A Saad EB Corrado A Gasparini G Marrouche NF Golshayan AR McCurdy R Bhargava M Khaykin Y Impact of coronary sinus lead position on biventricular pacing: mortality and echocardiographic evaluation during long-term follow-up J Cardiovasc Electrophysiol 2004 15 10 1120 1125 10.1046/j.1540-8167.2004.04089.x 15485432

[B66] Kronborg MB Albertsen AE Nielsen JC Mortensen PT Long-term clinical outcome and left ventricular lead position in cardiac resynchronization therapy Europace 2009 11 9 1177 1182 10.1093/europace/eup202 19661114

[B67] Ansalone G Giannantoni P Ricci R Doppler myocardial imaging in patients with heart failure receiving biventricular pacing treatment Am Heart J 2001 142 881 896 10.1067/mhj.2001.117324 11685178

[B68] Murphy RT Sigurdsson G Mulamalla S Agler D Popovic ZB Starling RC Wilkoff BL Thomas JD Grimm RA Tissue synchronization imaging and optimal left ventricular pacing site in cardiac resynchronization therapy Am J Cardiol 2006 97 11 1615 1621 10.1016/j.amjcard.2005.12.054 16728225

[B69] Becker M Hoffmann R Schmitz F Hundemer A Kuhl H Schauerte P Kelm M Franke A Relation of optimal lead positioning as defined by three-dimensional echocardiography to long-term benefit of cardiac resynchronization Am J Cardiol 2007 100 11 1671 1676 10.1016/j.amjcard.2007.07.019 18036367

[B70] Becker M Franke A Breithardt OA Ocklenburg C Kaminski T Kramann R Knackstedt C Stellbrink C Hanrath P Schauerte P Impact of left ventricular lead position on the efficacy of cardiac resynchronisation therapy: a two-dimensional strain echocardiography study Heart 2007 93 10 1197 1203 10.1136/hrt.2006.095612 17309913 PMC2000938

[B71] Rademakers L VanKerckhoven R vanDeursen C Strik M vanHunnik A Kuiper M Lampert A Klersy C Leyva F Auricchio A Myocardial Infarction Does Not Preclude Electrical and Hemodynamic Benefits of CRT in Dyssynchronous Canine Hearts Circ Arrhythm Electrophysiol 2010 in press 20495014 10.1161/CIRCEP.109.931865

[B72] Torrent-Guasp F Kocica MJ Corno AF Komeda M Carreras-Costa F Flotats A Cosin-Aguillar J Wen H Towards new understanding of the heart structure and function Eur J Cardiothorac Surg 2005 27 191 201 10.1016/j.ejcts.2004.11.026 15691670

[B73] Greenbaum RA Ho SY Gibson DG Becker AE Anderson RH Left ventricular fibre architecture in man Br Heart J 1981 45 3 248 263 10.1136/hrt.45.3.248 7008815 PMC482521

[B74] Yettram A Vinson CA Gibson DG Effect of myocardial fibre architecture on the behaviour of the human left ventricle in diastole J Biomed Eng 1983 5 321 328 10.1016/0141-5425(83)90008-0 6632844

[B75] Antzelevitch C Fish J Electrical heterogeneity within the ventricular wall Basic Res Cardiol 2001 96 517 527 10.1007/s003950170002 11770069

[B76] Cassidy D Vassallo J Miller J Poll D Buxton A Marchinski F Josephson M Endocardial catheter mapping in patients in sinus rhythm: relationship to underlying heart disease and ventricular arrhythmias Circulation 1986 73 645 652 3948367 10.1161/01.cir.73.4.645

[B77] Tournoux F Donal E Leclercq C De Place C Crocq C Solnon A Cohen-Solal A Mabo P Daubert JC Concordance Between Mechanical and Electrical Dyssynchrony in Heart Failure Patients: A Function of the Underlying Cardiomyopathy? J Cardiovasc Electrophysiol 2007 18 1022 1027 10.1111/j.1540-8167.2007.00900.x 17666067

[B78] Leclercq C Gadler F Kranig W Ellery S Gras D Lazarus A Clémenty J Boulogne E Daubert J TRIP-HF (Triple Resynchronization In Paced Heart Failure Patients) Study Group A randomized comparison of triple-site versus dual-site ventricular stimulation in patients with congestive heart failure J Am Coll Cardiol 2008 51 1455 1462 10.1016/j.jacc.2007.11.074 18402900

[B79] Allman KC Shaw LJ Hachamovitch R Udelson JE Myocardial viability testing and impact of revascularization on prognosis in patients with coronary artery disease and left ventricular dysfunction: a meta-analysis J Am Coll Cardiol 2002 39 1151 1158 10.1016/S0735-1097(02)01726-6 11923039

[B80] Wu E Judd RM Vargas JD Klocke FJ Bonow RO Kin RJ Visualisation of presence, location and transmural extent of healed Q-wave and non-Q-wave myocardial infarction Lancet 2001 357 21 28 10.1016/S0140-6736(00)03567-4 11197356

[B81] Kim RJ Wu E Rafael A Chen EL Parker MA Simonetti O Klocke FJ Bonow RO Judd RM The use of contrast-enhanced magnetic resonance imaging to identify reversible myocardial dysfunction N Engl J Med 2000 343 1445 1453 10.1056/NEJM200011163432003 11078769

[B82] Bellenger NG Yousef Z Kajappan K Marber MS Pennell DJ Infarct viability influences ventricular remodelling after late recanalisation of an occluded infarct related artery Heart 2005 91 478 483 10.1136/hrt.2004.034918 15772205 PMC1768832

[B83] Meluzin J Cerny J Frelich M Stetka F Spiranova F Popelova J Stipal R Prognostic value of the amount of dysfunctional but viable myocardium in revascularized patients with coronary artery disease and left ventricular dysfunction J Am Col Cardiol 1998 32 912 920 10.1016/S0735-1097(98)00324-6 9768711

[B84] Lee KS Marwick TH Cook SA Go RT Fix JS James KB Sapp SK McIntyre WJ Thomas JD Prognosis of patients with left ventricular dysfunction, with and without viable myocardium after myocardial infarction. Relative efficacy of medical therapy and revascularization Circulation 1994 90 2687 2694 7994809 10.1161/01.cir.90.6.2687

[B85] Bello D Shah DJ Farah GM Di Luzio S Parker MA Johnson M Cotts WG Klocke FJ Bonow RO Judd RM Gadolinium cardiovascular magnetic resonance predicts myocardial dysfunction and remodelling in patients with heart failure undergoing beta-blocker therapy Circulation 2003 108 1945 1953 10.1161/01.CIR.0000095029.57483.60 14557364

[B86] McCrohon JA Moon JJC Prasad SK McKenna WJ Lorenz CH Coats AJS Pennell DJ Differentiation of heart failure related to dilated cardiomyopathy and coronary artery disease using gadolinium-enhanced cardiovascular magnetic resonance Circulation 2003 108 54 59 10.1161/01.CIR.0000078641.19365.4C 12821550

[B87] Assomull RG Prasad SK Lyne J Smith G Burman ED Khan M Sheppard MN Poole-Wilson PA Pennell DJ Cardiovascular magnetic resonance, fibrosis, and prognosis in dilated cardiomyopathy J Am Coll Cardiol 2006 48 10 1977 1985 10.1016/j.jacc.2006.07.049 17112987

[B88] White JA Yee R Yuan X Krahn A Skanes A Parker M Klein G Drangova M Delayed enhancement magnetic resonance imaging predicts response to cardiac resynchronization therapy in patients with intraventricular dyssynchrony J Am Coll Cardiol 2006 48 10 1953 1960 10.1016/j.jacc.2006.07.046 17112984

[B89] Chalil S Foley P Muyhaldeen S Patel K Yousef Z Smith R Frenneaux M Leyva F Late gadolinium enhancement-cardiovascular magnetic resonance as a predictor of response to cardiac resynchronization therapy in patients with ischaemic cardiomyopathy Europace 2007 9 1031 1037 10.1093/europace/eum133 17933857

[B90] Adelstein EC Saba S Scar burden by myocardial perfusion imaging predicts echocardiographic response to cardiac resynchronization therapy in ischemic cardiomyopathy Am Heart J 2007 153 1 105 112 10.1016/j.ahj.2006.10.015 17174647

[B91] Schwartzman D Chang I Michele JJ Moritznik MS Foster KR Electrical impedance properties of normal and chronically infarcted ventricular myocardium J Interv Card Electrophysiol 1999 3 213 224 10.1023/A:1009887306055 10490477

[B92] Reddy VY Wrobleski D Houghtaling C Josephson ME Ruskin JN Combined Epicardial and Endocardial Electroanatomic Mapping in a Porcine Model of Healed Myocardial Infarction Circulation 2003 107 25 3236 3242 10.1161/01.CIR.0000074280.62478.E1 12796129

[B93] Tedrow U Maisel W Epstein L Soejima K Stevenson W Feasibility of adjusting paced left ventricular activation by manipulating stimulus strength J Am Coll Cardiol 2004 44 2249 2251 10.1016/j.jacc.2004.09.008 15582325

[B94] Breithardt OA Stellbrink C Kramer AP Sinha AM Franke A Salo R Schiffgens B Huvelle E Auricchio A PATH-CHF Study Group Echocardiographic quantification of left ventricular asynchrony predicts an acute hemodynamic benefit of cardiac resynchronization therapy J Am Coll Cardiol 2002 40 3 536 545 10.1016/S0735-1097(02)01987-3 12142123

[B95] Chalil S Stegemann B Muhyaldeen S Khadjooi S Foley P Smith R Leyva F Effect of posterolateral left ventricular scar on mortality and morbidity following cardiac resynchronization therapy Pacing Clin Electrophysiol 2007 10 1201 1207 10.1111/j.1540-8159.2007.00841.x 17897122

[B96] Moyé L Statistical reasoning in medicine 2006 New York, NY: Springer

[B97] Aaronson KD Schwartz JS Chen TM Wong KL Goin JE Mancini DM Development and prospective validation of a clinical index to predict survival in ambulatory patients referred for cardiac transplant evaluation Circulation 1997 95 12 2660 2667 9193435 10.1161/01.cir.95.12.2660

[B98] Lee DS Austin PC Rouleau JL Liu PP Naimark D Tu JV Predicting mortality among patients hospitalized for heart failure: derivation and validation of a clinical model JAMA 2003 290 19 2581 2587 10.1001/jama.290.19.2581 14625335

[B99] Anker S Doehner W Rauchhaus M Sharma R Francis D Knosalla C Davos C Cicoira M Shamim W Kemp M Uric acid and survival in chronic heart failure: validation and application in metabolic, functional, and hemodynamic staging Circulation 2003 107 15 1991 1997 10.1161/01.CIR.0000065637.10517.A0 12707250

[B100] Leyva F Foley PW Stegemann B Ward JA Ng LL Frenneaux MP Regoli F Smith RE Auricchio A Development and validation of a clinical index to predict survival after cardiac resynchronisation therapy Heart 2009 95 19 1619 1625 10.1136/hrt.2009.173880 19592389 PMC2735760

[B101] Dries DL Exner DV Domanski MJ Greenberg B Stevenson LW The prognostic implications of renal insufficiency in asymptomatic and symptomatic patients with left ventricular systolic dysfunction J Am Coll Cardiol 2000 35 3 681 689 10.1016/S0735-1097(99)00608-7 10716471

[B102] Smilde TD Hillege HL Navis G Boomsma F de Zeeuw D van Veldhuisen DJ Impaired renal function in patients with ischemic and nonischemic chronic heart failure: association with neurohormonal activation and survival Am Heart J 2004 148 1 165 172 10.1016/j.ahj.2004.02.007 15215807

[B103] Hillege HL Girbes AR de Kam PJ Boomsma F de Zeeuw D Charlesworth A Hampton JR van Veldhuisen DJ Renal function, neurohormonal activation, and survival in patients with chronic heart failure Circulation 2000 102 2 203 210 10889132 10.1161/01.cir.102.2.203

[B104] Levy WC Mozaffarian D Linker DT Sutradhar SC Anker SD Cropp AB Anand I Maggioni A Burton P Sullivan MD The Seattle Heart Failure Model: prediction of survival in heart failure Circulation 2006 113 11 1424 1433 10.1161/CIRCULATIONAHA.105.584102 16534009

[B105] Patel K Leyva F Frenneaux M Heart failure is not a diagnosis Int J Clin Pract 2008 62 526 528 10.1111/j.1742-1241.2008.01720.x 18324949

[B106] Wikstrom G Blomstrom-Lundqvist C Andren B Lonnerholm S Blomstrom P Freemantle N Remp T Cleland JG The effects of aetiology on outcome in patients treated with cardiac resynchronization therapy in the CARE-HF trial Eur Heart J 2009 30 7 782 788 10.1093/eurheartj/ehn577 19168870 PMC2663726

[B107] Alpert JS Thygesen K Antman E Bassand JP Myocardial infarction redefined--a consensus document of The Joint European Society of Cardiology/American College of Cardiology Committee for the redefinition of myocardial infarction J Am Coll Cardiol 2000 36 959 969 10.1016/S0735-1097(00)00804-4 10987628

[B108] Kannel WB Silent myocardial ischemia and infarction: insights from the Framingham Study Cardiology clinics 1986 4 4 583 591 3779719

[B109] McKenna WJ Chew CY Oakley CM Myocardial infarction with normal coronary angiogram. Possible mechanism of smoking risk in coronary artery disease Br Heart J 1980 43 5 493 498 10.1136/hrt.43.5.493 7378207 PMC482331

[B110] Assomull RG Lyne JC Keenan N Gulati A Bunce NH Davies SW Pennell DJ Prasad SK The role of cardiovascular magnetic resonance in patients presenting with chest pain, raised troponin, and unobstructed coronary arteries Eur Heart J 2007 28 10 1242 1249 10.1093/eurheartj/ehm113 17478458

[B111] Duncan AM Francis DP Gibson DG Henein MY Differentiation of ischemic from nonischemic cardiomyopathy during dobutamine stress by left ventricular long-axis function: additional effect of left bundle-branch block Circulation 2003 108 10 1214 1220 10.1161/01.CIR.0000087401.19332.B7 12939221

[B112] Kim RJ Fieno DS Parrish TB Harris K Chen EL Simonetti O Bundy J Finn JP Klocke FJ Judd RM Relationship of MRI delayed contrast enhancement to irreversible injury, infarct age, and contractile function Circulation 1999 100 19 1992 2002 10556226 10.1161/01.cir.100.19.1992

[B113] Carlsson M Arheden H Higgins CB Saeed M Magnetic resonance imaging as a potential gold standard for infarct quantification Journal of electrocardiology 2008 41 6 614 620 10.1016/j.jelectrocard.2008.06.010 18817927

[B114] Germans T van Rossum AC The use of cardiac magnetic resonance imaging to determine the aetiology of left ventricular disease and cardiomyopathy Heart 2008 94 4 510 518 10.1136/hrt.2007.122770 18347381

[B115] Modell B Khan M Darlison M Westwood MA Ingram D Pennell DJ Improved survival of thalassaemia major in the UK and relation to T2* cardiovascular magnetic resonance J Cardiovasc Magn Reson 2008 10 42 10.1186/1532-429X-10-42 18817553 PMC2563008

[B116] Giri S Chung YC Merchant A Mihai G Rajagopalan S Raman SV Simonetti OP T2 quantification for improved detection of myocardial edema J Cardiovasc Magn Reson 2009 11 56 10.1186/1532-429X-11-56 20042111 PMC2809052

[B117] Raman SV Simonetti OP Winner MW Dickerson JA He X Mazzaferri EL Jr Ambrosio G Cardiac magnetic resonance with edema imaging identifies myocardium at risk and predicts worse outcome in patients with non-ST-segment elevation acute coronary syndrome J Am Coll Cardiol 55 22 2480 2488 10.1016/j.jacc.2010.01.047 20510215 PMC3675879

[B118] Levine GN Gomes AS Arai AE Bluemke DA Flamm SD Kanal E Manning WJ Martin ET Smith JM Wilke N Safety of Magnetic Resonance Imaging in Patients With Cardiovascular Devices: An American Heart Association Scientific Statement From the Committee on Diagnostic and Interventional Cardiac Catheterization, Council on Clinical Cardiology, and the Council on Cardiovascular Radiology and Intervention: Endorsed by the American College of Cardiology Foundation, the North American Society for Cardiac Imaging, and the Society for Cardiovascular Magnetic Resonance Circulation 2007 116 24 2878 2891 10.1161/CIRCULATIONAHA.107.187256 18025533

[B119] Achenbach S Moshage W Diem B Bieberle T Schibgilla V Bachmann K Effects of magnetic resonance imaging on cardiac pacemakers and electrodes Am Heart J 1997 134 3 467 473 10.1016/S0002-8703(97)70083-8 9327704

[B120] Hayes DL Holmes DR Jr Gray JE Effect of 1.5 tesla nuclear magnetic resonance imaging scanner on implanted permanent pacemakers J Am Coll Cardiol 1987 10 4 782 786 10.1016/S0735-1097(87)80270-X 3655146

[B121] Pennell D Cardiac magnetic resonance with a pacemaker in-situ: can it be done? J Cardiovasc Magn Reson 1999 1 72

[B122] Martin ET Coman JA Shellock FG Pulling CC Fair R Jenkins K Magnetic resonance imaging and cardiac pacemaker safety at 1.5-Tesla J Am Coll Cardiol 2004 43 7 1315 1324 10.1016/j.jacc.2003.12.016 15063447

[B123] Schwartzman D Schelbert E Adelstein E Gorcsan J Soman P Saba S Image-guided cardiac resynchronization Europace 12 6 877 880 10.1093/europace/euq103 20400444

[B124] Chiribiri A Kelle S Gotze S Kriatselis C Thouet T Tangcharoen T Paetsch I Schnackenburg B Fleck E Nagel E Visualization of the cardiac venous system using cardiac magnetic resonance Am J Cardiol 2008 101 3 407 412 10.1016/j.amjcard.2007.08.049 18237610

[B125] Zwanenburg JJM Gotte MJW Kuijer JPA Heethaar RM van Rossum AC Marcus JT Timing of cardiac contraction in humans mapped by high-temporal-resolution MRI tagging: early onset and late peak of shortening in lateral wall Am J Physiol Heart Circ Physiol 2004 286 5 H1872 1880 10.1152/ajpheart.01047.2003 14726304

[B126] Dickstein K Vardas PE Auricchio A Daubert JC Linde C McMurray J Ponikowski P Priori SG Sutton R van Veldhuisen DJ 2010 Focused Update of ESC guidelines on device therapy in heart failure: An update of the 2008 ESC guidelines for the diagnosis and treatment of acute and chronic heart failure and the 2007 ESC guidelines for cardiac and resynchronization therapy Developed with the special contribution of the Heart Failure Association and the European Heart Rhythm Association Eur Heart J

[B127] Epstein AE DiMarco JP Ellenbogen KA Estes NA Freedman RA Gettes LS Gillinov AM Gregoratos G Hammill SC Hayes DL ACC/AHA/HRS 2008 Guidelines for Device-Based Therapy of Cardiac Rhythm Abnormalities: a report of the American College of Cardiology/American Heart Association Task Force on Practice Guidelines (Writing Committee to Revise the ACC/AHA/NASPE 2002 Guideline Update for Implantation of Cardiac Pacemakers and Antiarrhythmia Devices) developed in collaboration with the American Association for Thoracic Surgery and Society of Thoracic Surgeons J Am Coll Cardiol 2008 51 21 e1 62 10.1016/j.jacc.2008.02.032 18498951

[B128] Auricchio A Abraham WT Cardiac resynchronization therapy: Current state of the art: Cost versus benefit Circulation 2004 109 3 300 307 10.1161/01.CIR.0000115583.20268.E1 14744954

[B129] Lardo AC Abraham TP Kass DA Magnetic Resonance Imaging Assessment of Ventricular Dyssynchrony: Current and Emerging Concepts J Am Coll Cardiol 2005 46 12 2223 2228 10.1016/j.jacc.2005.09.015 16360050

[B130] Leyva F Foley PWX Stegemann B Ward JA Ng LL Frenneaux MP Regoli F Smith REA Auricchio A Development and validation of a clinical index to predict survival after cardiac resynchronisation therapy Heart 2009 95 19 1619 1625 10.1136/hrt.2009.173880 19592389 PMC2735760

